# A new species of terrestrial frog *Pristimantis* (Strabomantidae) from the upper basin of the Pastaza River, Ecuador

**DOI:** 10.3897/zookeys.832.30874

**Published:** 2019-03-19

**Authors:** Carolina Reyes-Puig, Juan Pablo Reyes-Puig, Daniel A. Velarde-Garcéz, Nicolás Dávalos, Emilio Mancero, María José Navarrete, Mario H. Yánez-Muñoz, Diego F. Cisneros-Heredia, Santiago R. Ron

**Affiliations:** 1 Instituto de Zoología Terrestre & Museo de Zoología, Universidad San Francisco de Quito USFQ, Colegio de Ciencias Biológicas y Ambientales, Quito, Ecuador; 2 Unidad de Investigación, Instituto Nacional de Biodiversidad (INABIO), Quito, Ecuador; 3 Fundación Red de Protección de Bosques ECOMINGA, Fundación Oscar Efrén Reyes, Departamento de Ambiente, Baños, Ecuador; 4 Museo de Zoología, Escuela de Ciencias Biológicas, Pontificia Universidad Católica del Ecuador, Quito, Ecuador; 5 King’s College London, Department of Geography, London, UK

**Keywords:** Montane forest, *Pristimantismallii* sp. n., Río Zuñag Reserve, Terrarana

## Abstract

We describe a new species of *Pristimantis* from the montane forest of the Río Zuñag Ecological Reserve, upper basin of the Pastaza River, Ecuador. *Pristimantismallii***sp. n.** is characterized by a snout-vent length of 11.6–21.3 mm in adult males (*n* = 12), 22.6–34.3 mm in adult females (*n* = 8), and is compared morphologically and genetically with *Pristimantismiktos* and with other relevant species of *Pristimantis*. The new species is characterized by having skin on dorsum and flanks shagreen, distinctive scapular folds, snout broadly rounded in dorsal view, upper eyelid bearing one or two subconical tubercles and some rounded tubercles, dorsum and flanks light brown to brown, with irregular dark brown marks bounded by dirty cream and groin with irregular yellowish marks.

## Abstract (es)

Describimos una nueva especie de *Pristimantis* del bosque montano de la Reserva Ecológica Río Zuñag, cuenca alta del río Pastaza, Ecuador. *Pristimantismallii***sp. n** es caracterizada por una longitud rostro-cloacal de 11.6–21.3 mm en machos adultos (*n* = 12), 22.6–34.3 mm en hembras adultas (*n* = 8), y es comparada morfológica y genéticamente con *Pristimantismiktos* y con otras especies relevantes de *Pristimantis*. La especie nueva se caracteriza por tener la piel del dorso y flancos finamente granular, pliegues escapulares distintivos, hocico redondeado en vista dorsal, párpado superior con uno dos tubérculos subcónicos y algunos redondeados, dorso y flancos café claro a café, con marcas irregulares café oscuras bordeadas de crema sucio y marcas irregulares amarillentas en las ingles.

## Introduction

The genus *Pristimantis* is an endemic group of terrestrial frogs of the Neotropical region; with more than 525 species, it is the largest genus of all vertebrates (Frost 2018). Its highest diversity is found in the Andean montane forests of Colombia, Ecuador, and Peru (Heinicke et al. 2007; Frost 2018). In Ecuador, this genus represents 38.5% of the amphibians, with 230 species currently described, of which 125 are endemic (Ron et al. 2018). In the last five years, 37 species of *Pristimantis* in Ecuador have been described (Ron et al. 2018). This rapid and continuous increase of the known species suggests that this number will keep rising, considering the many regions that still remain unexplored in the Ecuadorian Andes. Presumably, the high diversity of this terrestrial group is explained by the success of their direct development, which allows individuals to be independent of water and to colonize new terrestrial niches (Hedges et al. 2008). Most of the species of this genus are characterized by having small distributions (Lynch and Duellman 1980; Terán-Valdez and Guayasamin 2010; Yánez-Muñoz et al. 2016). This could explain the high rate of discovery in the eastern Andes of Ecuador, where recently several species have been described (e.g. Reyes-Puig et al. 2010, 2013, 2014; Reyes-Puig and Yánez-Muñoz 2012; Batallas and Brito 2014; Brito et al. 2014, 2016, 2017a, b; Yánez-Muñoz et al. 2014; Navarrete et al. 2016).

The upper basin of the Pastaza River is an important endemic region, mainly because the Río Pastaza is a major Ecuadorian tributary of the Amazon (i.e. biogeographic barrier), with a rugged topography of volcanic and granitic origin (Kennerly and Bromley 1971; Gradstein et al. 2004; Sánchez et al. 2018). These characteristics have allowed the presence of flora and fauna distributed only in this restricted region (e.g. Gradstein et al. 2004; Reyes-Puig et al. 2010, 2013, 2014, 2015; Jost and Shepard 2017). Here we describe a new species of direct-developing frog of the genus *Pristimantis* from the montane forest of the Pastaza River basin, with morphological and phylogenetic analyzes based on lab and field work executed by several institutions.

## Materials and methods

### DNA extraction, amplification and sequencing

DNA extraction and amplification processes took place at the Laboratorio de Biología Molecular of the Museo de Zoología, Pontificia Universidad Católica del Ecuador (QCAZ). Total DNA was extracted from liver and muscular tissue preserved in 95% ethanol by applying the Guanidinium thiocyanate (GITC) protocol (Esselstyn et al. 2008). Samples were quantified on a nanodrop (Thermo Scientific) and diluted by aliquots at a 20 ng/µl concentration. Standard PCR procedures were used to amplify the mitochondrial gene 16S rRNA (16S) and the nuclear recombination-activating genes (RAG1). The primers used were 16L19 and 16H36E for 16S (Heinicke et al. 2007) as well as RAG1FF2 and RAG1FR2 for RAG (Heinicke et al. 2007). The amplified results were purified by the ExoSap tool and sent to the Macrogen company (Macrogen Inc., Seoul, Korea) for sequencing. Additionally, the genetic sample included various 12S rRNA (12S) mitochondrial gene sequences obtained from the GenBank database.

The sequences generated de novo were assembled and edited manually on the GeneiousPro 5.4.6 software (Biomatters Ltd). Both ends of the sequence were cut during editing to avoid low quality base pairs. GenBank Access codes were assigned to new sequences presented in this study are MK391384, RAG1; MK391386, 16S, tRNAs, ND1 for QCAZ 52473 (*Pristimantismallii* sp. n.). MK391383, RAG1; MK391385, 16S, tRNAs, ND1 for QCAZ 55445 (*Pristimantismiktos*).

### Phylogenetic Analysis

New sequences were compared to the GenBank sequences using the BLAST tool (http://blast.ncbi.nlm.nih.gov/Blast.cgi) in order to confirm their genetic identity and determine similar species that allow the evaluation of the phylogenetic position of the new taxon. The search showed a high likeness between the new species and *Pristimantisriveti*. Therefore, we have included comparisons with *P.riveti* and other closely related species (sensu Padial et al. 2014), as well as other representative species from the *Pristimantis* clade. Sequences from *Diasporus*, *Eleutherodactylus*, *Holoaden*, *Hypodactylus, Ischnocnema*, *Lynchius*, *Oreobates*, and *Strabomantis* were also used as external groups. GenBank sequences employed correspond to data previously reported by Darst and Cannatella (2004), Faivovich et al. (2005), Elmer et al. (2007), Heinicke et al. (2007, 2009, 2015), Hedges et al. (2008), Arteaga-Navarro and Guayasamin (2011), Fouquet et al. (2012), Kok et al. (2012, 2018), Lehr et al. (2012, 2017), Lehr and Von May (2017), Pinto-Sánchez et al. (2012), Arteaga et al. (2013, 2016), Barrio-Amorós et al. (2013), Crawford et al. (2013), Zhang et al. (2013), Ortega-Andrade and Venegas (2014), Rivera-Prieto et al. (2014), Hutter and Guayasamin (2015), Rivera-Correa and Daza (2015), Chávez and Catenazzi (2016), de Oliveira et al. (2017), García-R. et al. (2012), Shepack et al. (2016), Székely et al. (2016), González-Durán et al. (2017), Guayasamin et al. 2015, Guayasamin et al. 2017, Jablonski et al. (2017), von May et al. (2017), Mahecha et al. (unpub.).

Multiple sequence alignment was done on the GeneiousPro 5.4.6 software under the MUSCLE algorithm (Robert 2004). Revision and manual correction of the matrix was performed on the Mesquite v2.75 software (Maddison and Maddison 2011). The codifying loci (RAG) were translated in amino acids to evaluate and avoid the presence of stop codons. In total, the combined DNA matrix showed 2968 base pairs. The best model for trait evolution and the best partition outline for our data were estimated simultaneously in the PartitionFinder v1.1.1 software (Lanfear et al. 2012), by means of five partitions of the *a priori* configured matrix: one for 12S, one for 16S and one partition for each RAG codon.

Phylogenetic trees were rebuilt based on Bayesian inference and maximum likelihood estimation (MLE). For the MLE analyses, 4 independent searches of one replica each were performed, two of them under the systematized starting command stepwise (streefname = stepwise) and the remaining two were configured under the alternative command random (streefname = random). Phylogenetic searches ended after 2000000 degenerations with no improvement in the tree’s topology (genthreshfortopoterm = 2000000). The support of each branch was estimated considering 200 bootstrap replicas obtained under the same configuration parameters used to determine the best tree. The consensus tree was estimated in the Mesquite v2.75 software by a 50% majority consensus rule. Bayesian inference analyses took place on the Mrbayes v3.2 software (Ronquist et al. 2012) available online on the CIPRES Science Gateway portal (Miller et al. 2010). The search consisted of five parallel runs of Markov Monte Carlo chain, each one configured at 20 × 106 search generations, four chains at standard temperature values. 50% of the generations were removed as burn-in. By using the TRACER v1.6 (Rambaut and Drummond 2007) software we confirmed the convergence in our searches, with an effective stationary distribution and sample size (ESS > 200). Finally, non-corrected *p* genetic distances were estimated based on the 16S gene for the new species and related clades using the Mega 6 (Tamura et al. 2013) software. The reference threshold for genetic separation used in the present study is 3% to determine different species (Fouquet et al. 2007).

### Morphological data

Description, measurements and terminology follow the standardized format of Lynch and Duellman (1997). The diagnostic characters follow the definitions of Duellman and Lehr (2009). The collected specimens were sacrificed with lidocaine, fixed in 10% formalin and preserved in 70% ethanol. The sex and age of the specimens were determined by secondary sexual characteristics (nuptial pads, vocal slits and size) and direct inspection of the gonads through a dorsolateral incision. The following measurements were taken by the same person at least three times and were averaged with calipers to the nearest 0.1 mm: snout-vent length (SVL), tibia length (TL), foot length (FL), head width (HW), head length (HL), interorbital distance (IOD), width of the upper eyelid (EW), internarial distance (IND), eye-nostril distance (EN), tympanum diameter (TD), eye diameter (ED). The life coloration pattern of the specimens was recorded with field notes and in-field color photography. The localities, coordinates and elevations were determined from field notes of the collectors and taken with a GPS receiver. The examined specimens were deposited in the Museo de Zoología, MUniversidad San Francisco de Quito (ZSFQ); Museo de Zoología, Pontificia Universidad Católica del Ecuador (QCAZ); and Sección de Herpetología, Instituto Nacional de Biodiversidad (DHMECN). All institutions are located in Quito, Ecuador.

## Results

### Phylogenetic relationships and genetic distances (Fig. 1)

**Figure 1.**
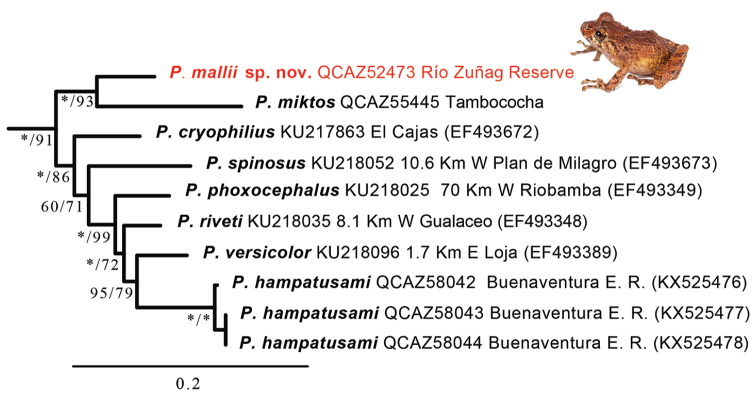
Phylogeny of *Pristimantis* showing the relationships of *Pristimantismallii* sp. n. (red). The phylogram was derived from analysis of 2968 bp of mitochondrial (gene fragments 12S and 16S) and nuclear (gene fragment RAG) DNA sequences. Branch support is presented for each clade as Bayesian posterior probabilities × 100 (left of the slash) and non-parametric bootstrap (right of the slash). Asterisks indicate support values of 100. The external group is not shown. For each specimen, museum catalog number, locality, and GenBank accession number (in parentheses) are reported. Abbreviations: E. R. = Ecological Reserve.

Placement of *Pristimantismallii* sp. n. in the genus *Pristimantis* was strongly supported, and according to the available information, the new species is sister to *Pristimantismiktos*. Both species form a clade with high support (Fig. 1) sister to a clade composed of *Pristimantiscryophilius*, *P.spinosus*, *P.phoxocephalus*, *P.riveti*, *P.versicolor*, and *P.hampatusami*. The uncorrected *p*-genetic distance between the new species and *P.miktos* is 11.9% (gene16S).

### Systematic accounts

#### Pristimantis mallii sp. n.

Taxon classificationAnimaliaAnuraStrabomantidae

http://zoobank.org/6B898DBA-743A-470A-ABC3-7B123648DFB5

**Figure 2.**
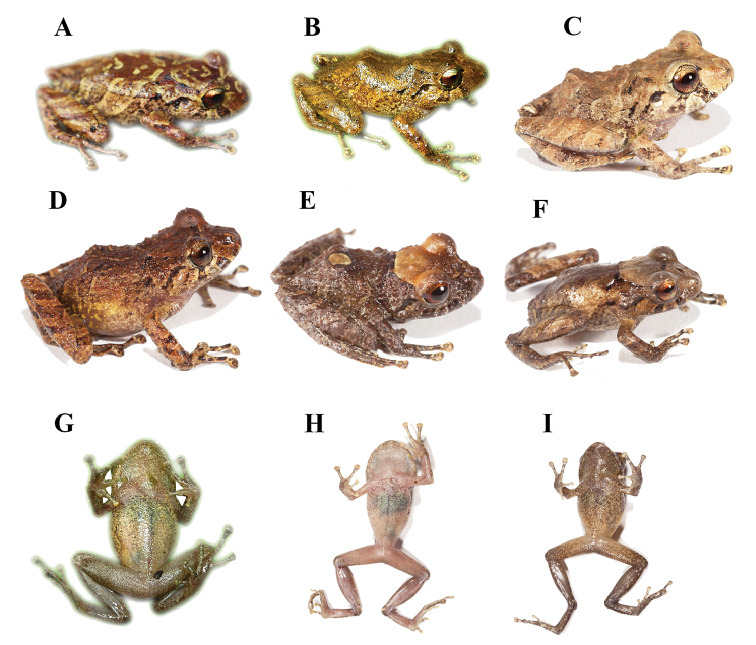
Coloration in life of *Pristimantismallii* sp. n. Dorsal view. **A**ZSFQ 1305, SVL = 34.3 mm, adult female **B**DHMECN 5236, SVL = 30.9 mm, adult female **C**QCAZ 52473, SVL = 28.8 mm, holotype, adult female; Second line from left to right **D**QCAZ 52494, SVL = 29.3 mm, adult female **E**QCAZ 52512, SVL = 10.3 mm, subadult male **F**QCAZ 52474, SVL = 11.6 mm, adult male. Ventral view **G**DHMECN 5236, SVL = 30.9 mm, adult female **H**QCAZ 52473, SVL = 28.8 mm, holotype, adult female **I**QCAZ 52474, SVL = 11.6 mm, adult male. Pictures are not to scale.

**Figure 3.**
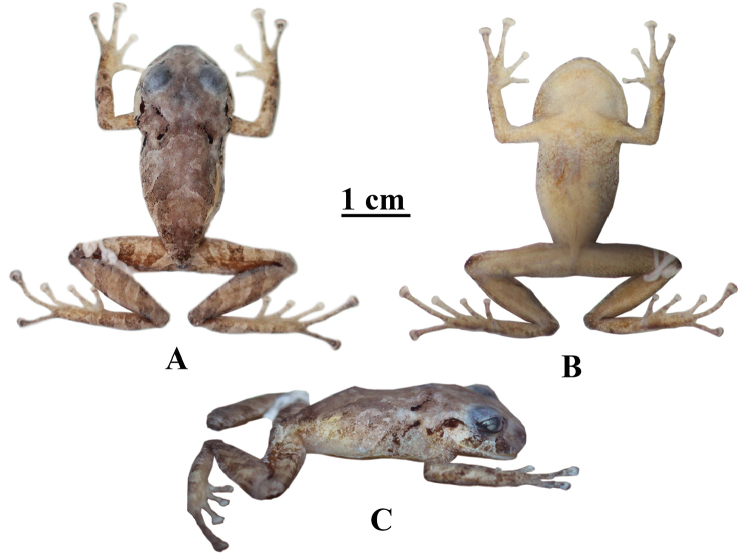
Preserved holotype of *Pristimantismallii* sp. n., QCAZ 52473, adult female, SVL = 28.8 mm **A** dorsal view **B** ventral view **C** lateral view.

**Figure 4.**
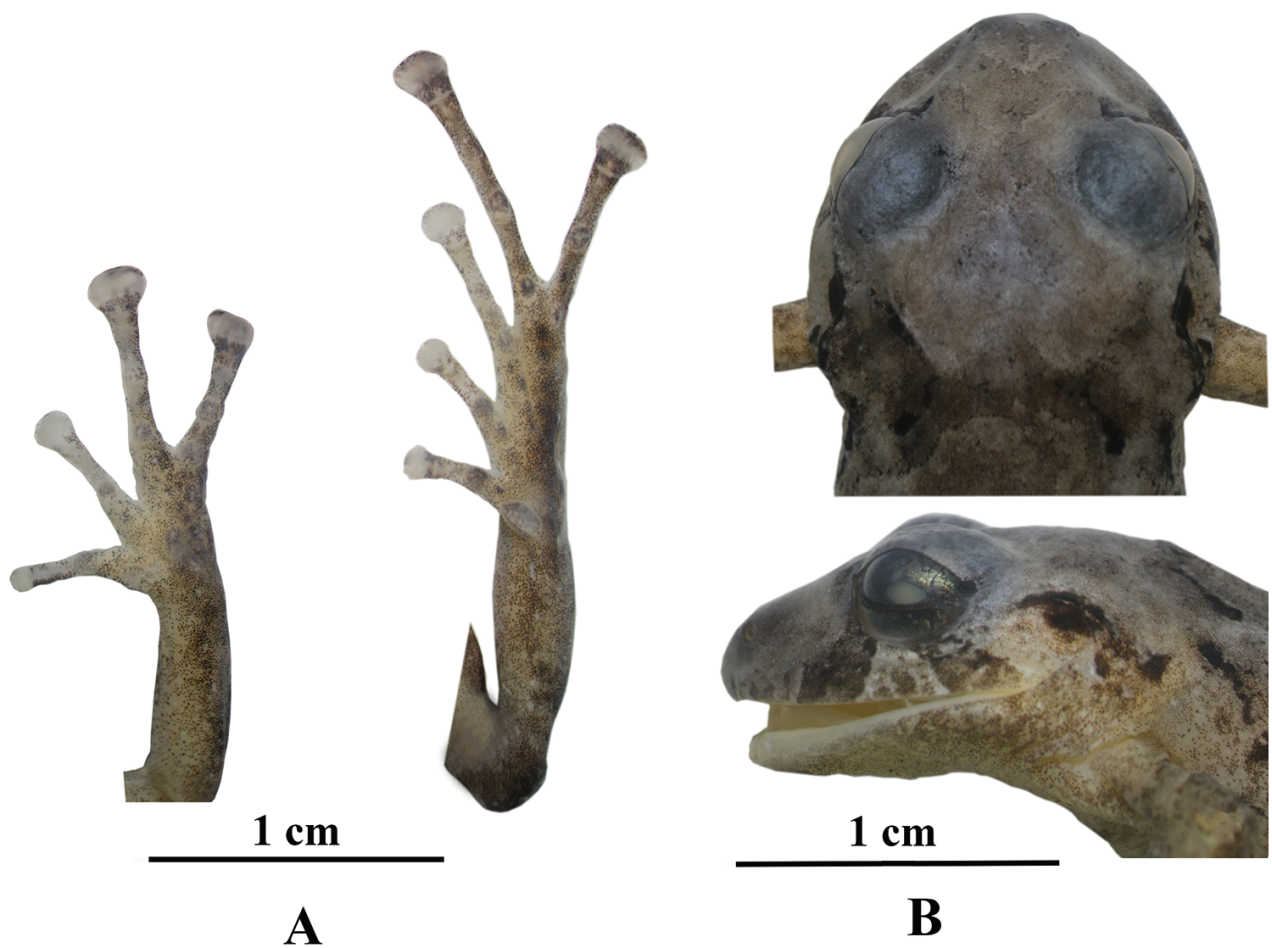
Preserved holotype of *Pristimantismallii* sp. n., QCAZ 52473, adult female, SVL = 28.8 mm **A** palmar and plantar surfaces **B** dorsal and lateral views of the head.

**Figure 5.**
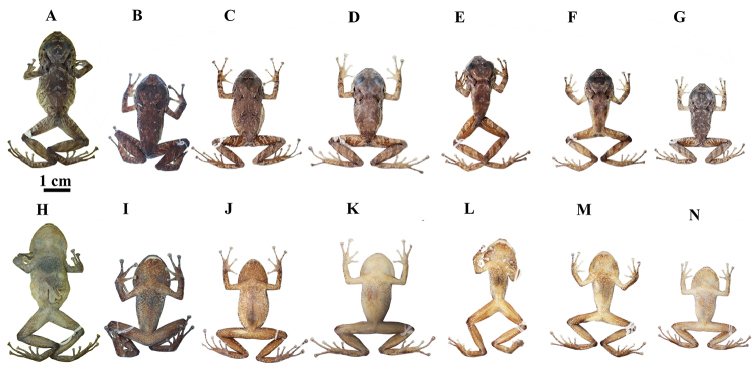
Preserved individuals of *Pristimantismallii* sp. n. showing dorsal and ventral variation in adult females **A–G** dorsal view **H–N** ventral view. **A, H**ZSFQ 1305, SVL = 34.3 mm **B, I**DHMECN 5236, SVL = 30.9 mm **C, J**QCAZ 52494, SVL = 29.3 mm **D, K**QCAZ 52473, SVL = 28.8 mm, holotype **E, L**QCAZ 39777, SVL = 26.5 mm **F, M**QCAZ 52477, SVL= 24.7 mm **G, N**QCAZ 52476, SVL = 24.0 mm.

**Figure 6.**
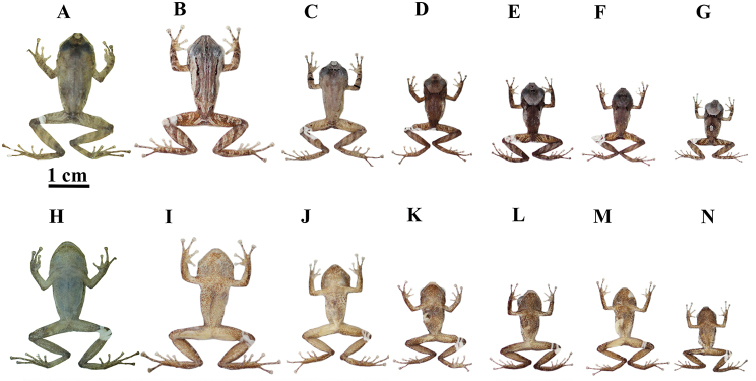
Preserved individuals of *Pristimantismallii* sp. n. showing dorsal and ventral variation in males **A–G** dorsal view **H–N** ventral view. **A, H**ZSFQ 1306, SVL = 21.3 mm **B, I**QCAZ 52480, SVL = 21.1 mm **C, J**QCAZ 52481, SVL 15.6 = mm **D, K**QCAZ 52471, SVL = 12.9 mm **E, L**QCAZ 52474, SVL = 11.6 mm **F, M**QCAZ 52478, SVL = 12.2 mm **G, N**QCAZ 52512, SVL = 10.3 mm.

Figures 2 , 3 , 4 , 5 , 6

##### Holotype.

QCAZ 52473 (field no. SC-PUCE 35222; Figs 3, 4), adult female, collected by Fernando Ayala, Diego Paucar, Yerka Sagredo, Juan Pablo Reyes-Puig, Fausto Recalde, Luis Recalde and Santiago Recalde on January 17, 2012 at Reserva Río Zuñag, Fundación EcoMinga, Baños, province of Tungurahua, Ecuador (1.36740S, 78.14573W; 2140 m elev.).

##### Paratypes

(7 females, 12 males). QCAZ 39777, adult female, collected by Diego Páez on January 1, 2009 at Reserva Río Zuñag, Fundación EcoMinga, Baños, province of Tungurahua, Ecuador (1.349399S, 78.15870W; 2127 m elev.). QCAZ 52476, 52477, adult females, collected by Fernando Ayala, Diego Paucar, Yerka Sagredo, Juan Pablo Reyes-Puig, Fausto Recalde, Luis Recalde and Santiago Recalde on January 17, 2012 at Reserva Río Zuñag, Fundación EcoMinga, Baños, province of Tungurahua, Ecuador (1.36761S, 78.14584W; 2153 m elev.). QCAZ 52494, adult female, collected by Fernando Ayala, Diego Paucar, Yerka Sagredo, Juan Pablo Reyes-Puig, Fausto Recalde, Luis Recalde and Santiago Recalde on January 17, 2012 at Reserva Río Zuñag, Fundación EcoMinga, Baños, province of Tungurahua, Ecuador (1.37220S, 78.15386W; 1823 m elev.). DHMECN 5236, 5264, adult females, collected by Mario Yánez-Muñoz, Miguel Urgilés and Andrés Laguna on May, 2008 at Reserva Río Zuñag, Baños, Province of Tungurahua, Ecuador (1.40045S, 78.186776W; 1300 m). ZSFQ 1305, adult female, collected by Carolina Reyes-Puig, Nicolás Dávalos, Daniel Velarde and Emilio Mancero on October 7, 2017 at at Reserva Río Zuñag, Fundación EcoMinga, Baños, province of Tungurahua, Ecuador (1.36761S, 78.14583W; 2190 m elev.). QCAZ 52512, subadult male, collected by Fernando Ayala, Diego Paucar, Yerka Sagredo, Juan Pablo Reyes-Puig, Fausto Recalde, Luis Recalde and Santiago Recalde on January 20, 2012 at Reserva Río Zuñag, Fundación EcoMinga, Baños, province of Tungurahua, Ecuador (1.37513S, 78.16363W; 1532 m elev.). QCAZ 52471, 52474, adult males with the same data of the holotype. QCAZ 52478, adult male, collected by Fernando Ayala, Diego Paucar, Yerka Sagredo, Juan Pablo Reyes-Puig, Fausto Recalde, Luis Recalde and Santiago Recalde on January 17, 2012 at Reserva Río Zuñag, Fundación EcoMinga, Baños, province of Tungurahua, Ecuador (1.36761S, 78.14583W; 2146 m elev.). QCAZ 52480, 52481, adult males, collected by Fernando Ayala, Diego Paucar, Yerka Sagredo, Juan Pablo Reyes-Puig, Fausto Recalde, Luis Recalde and Santiago Recalde on January 17, 2012 at Reserva Río Zuñag, Fundación EcoMinga, Baños, province of Tungurahua, Ecuador (1.36765S, 78.14594W; 2135 m elev.). DHMECN 5233–5235, 5238, adult males, collected by Mario Yánez-Muñoz, Miguel Urgiles and Andrés Laguna on May, 2008 at Reserva Río Zuñag, Baños, Province of Tungurahua, Ecuador (1.40045S, 78.186776W; 1269 m elev.). ZSFQ 1306, 1327, adult males, collected by Carolina Reyes-Puig, Nicolás Dávalos, Daniel Velarde and Emilio Mancero on October 7, 2017 at Reserva Río Zuñag, Fundación EcoMinga, Baños, province of Tungurahua, Ecuador (1.36761S, 78.14583W; 2190 m elev.).

##### Generic placement.

We assign the new species in *Pristimantis* based on our molecular data (Fig. 1).

##### Diagnosis.

A new species of *Pristimantis* having the following combination of characters: (1) skin on dorsum and flanks shagreen, with rounded tubercles scattered towards the axillary region, with “) (” shaped scapular folds (evident in life); dorsolateral folds absent; skin on venter areolate; discoidal fold slightly defined; (2) tympanic membrane and tympanic annulus present, round, equivalent to 45% of ED; supratympanic fold present; (3) snout broadly rounded in dorsal view, moderate in length and rounded in lateral view; (4) upper eyelid with one or two subconical tubercles on the center of eyelid and some rounded tubercles (less evident in preserved specimens); EW 100% of IOD; cranial crests absent (5) dentigerous processes of vomers oblique in outline, with five to seven teeth, moderately separated, posteromedial to choanae; (6) vocals slits and nuptial pads present; (7) Finger I shorter that Finger II; discs of digits expanded, truncate; two times the width of the digits on Fingers III and IV; (8) fingers with lateral fringes; (9) ulnar tubercles present, rounded; (10) heel bearing one or two subconical tubercles (less evident in preserved specimens) surrounded by few lower rounded tubercles; inner tarsal fold present, it extends up to 1/4 of the tarsus; (11) inner metatarsal tubercle oval, 5–6× as large as outer metatarsal tubercle that is subconical; supernumerary plantar tubercles indistinct; (12) toes with slightly defined lateral fringes; webbing absent; Toe V longer that Toe III, disc on Toe V reach the distal subarticular tubercle on Toe IV; (13) in life, dorsum and flanks light brown to brown, with irregular dark brown marks bounded by dirty cream, light brown or greenish cream; hidden surfaces of thighs brown splashed with dirty cream; groin with irregular yellowish marks; venter light gray or cream, spotted to densely spotted with brown. Golden coppery iris with black reticulations and a reddish-brown horizontal stripe; (14) SVL in adult males 16.7 ± 4.5 (11.6–21.3 mm); females with 27.6 ± 3.9 (22.6–34.3).

##### Comparisons with other species.

*Pristimantismallii* is most similar to its sister species *P.miktos* (Ortega-Andrade and Venegas 2014) (characters in parentheses) from the eastern lowlands of Ecuador. However, *P.mallii* can be easily distinguished for having “) (” shaped scapular folds (W- or X-shaped scapular fold); snout broadly rounded in dorsal view (subacuminate); upper eyelid bearing one or two subconical tubercles and some rounded tubercles (one small non-conical tubercle); dentigerous processes of vomers with 5–7 teeth (2 or 3 teeth); vocal slits in males present (absent); lateral fringes present (absent); dorsum and flanks light brown to brown, with irregular dark brown marks bounded by dirty cream, light brown or greenish cream (dorsum reddish-brown with some greenish-orange stains in scapular region, with or without yellowish-pale spots); golden coppery iris with black reticulations and a reddish-brown horizontal stripe (deep orange finely reticulated with black).

Other species of *Pristimantis* from the eastern lowlands of Ecuador, that can be confused with the new species by having dermal ridges in the scapular region, are *P.kichwarum* (Elmer and Cannatella 2008) and *P.luscombei* (Duellman and Mendelson 1995). Nonetheless, these two species have W-shaped dermal ridges (“) (” shaped fold in *P.mallii*); snout subacuminate in dorsal view (broadly rounded in *P.mallii*); ulnar tubercles absent or low (present, rounded in *P.mallii*); and nuptial pads in males absent (present in *P.mallii*). Other species of rain frogs from the eastern Andean slopes of Ecuador that are morphologically similar to *Pristimantismallii* are *P.marcoreyesi* (Reyes-Puig et al., 2014), *P.yanezi* (Navarrete et al., 2016) and *P.spinosus* (Lynch, 1979). In males of *Pristimantismarcoreyesi*, *P.yanezi* and *P.spinosus* the vocal slits and nuptial pads are absent (present in *P.mallii*). The snout in *P.marcoreyesi* and *P.spinosus* is subacuminate in dorsal view (broadly rounded in *P.mallii*); furthermore, *P.marcoreyesi* has dorsolateral folds slightly defined (absent in *P.mallii*), *P.spinosus* has the skin of dorsum finely tuberculate (shagreen in *P.mallii*), and the groin is black enclosing white spots (groin with irregular yellowish marks in *P.mallii*). Besides, *P.yanezi* can be distinguished by having one conical tubercle on the upper eyelid (one or two subconical in *P.mallii*); discoidal fold absent (present, slightly defined in *P.mallii*); fingers and toes without lateral fringes (present in *P.mallii*); dorsum yellowish brown to dark brown with scattered pale brown or orange blotches and black flecks, bearing a faint mid-dorsal hourglass-shaped band (dorsum and flanks light brown to brown, with irregular dark brown marks bounded by dirty cream, light brown or greenish cream).

##### Description of the holotype.

Adult female. Measurements (in mm): SVL 28.7; tibia length 15.6; foot length 15.2; head length 11.7; head width 12.8; eye diameter 4.7; tympanum diameter 2.5; interorbital distance 3.6; upper eyelid width 3.7; internarial distance 3.5; eye–nostril distance 4.6. Head slightly wider than long (12.8 mm vs 11.7); head width 44.4% of SVL; head length 41% of SVL; snout broadly rounded in dorsal view, moderate in length and rounded in lateral view (Fig. 4); eye–nostril distance 16% of SVL; canthus rostralis slightly concave in lateral view, nostrils directed laterally; interorbital area flat, as wide as the upper eyelid; cranial crests absent; upper eyelid with one or two subconical tubercles (reduced by preservation effects), and some rounded tubercles; upper eyelid width 100% of IOD; tympanic membrane differentiated, tympanic annulus present, with upper margins covered by a supratympanic fold; tympanum diameter 54% of eye diameter; three subconical postrictal tubercles. Choanae moderately in size, with a drop-shaped outline, not concealed by palatal shelf of maxilla; dentigerous processes of vomer oblique in outline, moderately separated, posteromedial to choanae, with six to seven teeth; tongue wider than long, notched posteriorly, approximately 40% of it fixed to the mouth floor.

Skin on dorsum and flanks shagreen, with rounded tubercles scattered towards the axillary region, with “) (” shaped scapular folds (evident in life); dorsolateral folds absent; skin on venter areolate; discoidal fold slightly defined; cloaca with rounded tubercles on the inferoposterior margin. Forearms slender, ulnar tubercles present, rounded; palmar tubercle heart-shaped, bilobed, approximately twice the size of oval thenar tubercle (the tubercles are slightly defined); subarticular tubercles rounded, defined, few supernumerary tubercles, indistinct; fingers with narrow lateral fringes; Finger I shorter than Finger II; disc on Finger I rounded and on Finger II expanded, twice the width of the digits on Fingers III and IV, truncate; pads on fingers well defined by circumferential grooves on all fingers (Fig. 4).

Hindlimbs slender, tibia length 54% of SVL; foot length 53% of SVL heel; upper surfaces of hindlimbs shagreen; posterior surfaces of thighs smooth, ventral surfaces areolate; heel bearing one or two subconical tubercles (less evident by preservation effect) surrounded by few lower rounded tubercles; inner tarsal fold present, it extends up to 1/4 of the tarsus; inner metatarsal tubercle oval, 5–6× as large as outer metatarsal tubercle that is subconical; supernumerary plantar tubercles indistinct; toes with slightly defined lateral fringes; webbing absent; discs on Fingers I and II rounded, discs in Fingers III, IV and V expanded, twice the width of the digit; all toes with ventral pads well defined by circumferential grooves; Toe V longer that Toe III, disc on Toe V reach the distal subarticular tubercle on Toe IV (Fig. 4).

##### Color of holotype in life.

(based on digital photographs, Fig. 2) Dorsum light brown with irregular brown marks bounded by dirty cream; black “) (” shaped scapular fold; head with dirty cream marks, one located behind the nostrils; flanks with brown oblique stripes delineated of dirty cream, with marbled brown marks concentrated towards axillary region; with two diagonal dark brown subocular stripes. Ventral areas of body, limbs and palms cream spotted with minute brown dots; throat cream spotted with brown dots, aggregates towards the outer edge of the jaw; forearms and hindlimbs with transversal brown bars separated by light brown interspaces; posterior surfaces of thigh dark brown; groin with irregular pale-yellowish marks. Golden coppery iris with black reticulations and a reddish-brown horizontal stripe.

##### Color of holotype in ethanol 70%.

(Fig. 3) Dorsum light brown with irregular brown marks slightly bounded by cream; black “) (” shaped scapular fold; with two black longitudinal lines above and behind the tympanum; with two diagonal brown subocular stripes; dorsal surfaces limbs, fingers and toes with transversal brown bars separated by dirty cream; the anterior surfaces of flanks light brown, with marbled brown marks concentrated towards axillary region; posterior surfaces of flanks and groin grayish cream. Ventral areas of body, limbs and palms cream spotted with minute brown dots aggregates towards the outer edge of the jaw, carpus, ulnar surfaces, flanks, posterior surfaces of thigh and tarsus. Golden olive iris.

##### Variation.

**Preserved individuals** (Figs 5, 6). In the type series, adult males (10.2–21.3 mm) are smaller than females (22.6–34.3). See Table 1 for measurements of the type specimens. Males have vocals slits located in the posteromedial region of the floor of the mouth; and nuptial pads located in the lower external portion of the Finger I. The “) (” shaped scapular fold is present in all individuals, but is black in all females, while it is not in some males (ZSFQ 1306, QCAZ 52481) (Figs 5, 6). Background coloration varies from gray or light brown to brown. Marks on dorsum and flanks are similar in all the type series, except for the adult males (ZSFQ 1306, QCAZ 52481) that have a dorsum without irregular marks and exhibit an internarial cream brand (Fig. 6). Some males present a gray patch on the head between the narinal and postorbital region (QCAZ 52471, QCAZ 52474, QCAZ 52478, and QCAZ 52512); this pattern is not present in any female (Figs 5, 6). The males (QCAZ 52512 and DHMECN 5234) have a gray spot in the middorsal region (Fig. 6). One male presents a dorsal pattern with longitudinal stripes on the dorsum (QCAZ 52480) (Fig. 6). In general, males have more variable dorsal patterns than females. Ventral coloration varies from cream to light brown; from slightly spotted (ZSFQ 1305, QCAZ 52473) to roughly spotted with brown (DHMECN 5236, QCAZ 52474, QCAZ 52212) (Figs 5, 6).

**Table 1.** Measurements (in mm) of type series of *Pristimantismallii* sp. n. Ranges followed by mean and standard deviation in parentheses.

Characters	Females (*n* = 8)	Males (*n* = 12)
**SVL**	22.6–34.3 (27.6±3.9)	10.2–21.3 (16.7±4.5)
**TL**	13.1–16.0 (15.3±1.0)	8.8–11.4 (10.7±1.0)
**FL**	12.2–15.0 (14.3±1.2)	8.2–11.4 (9.6±1.1)
**HW**	9.4–14 (11.8±1.4)	5.8–8.9 (7.4±0.9)
**HL**	9.5–14.2 (12.1±1.4)	6.2–8.2 (8.1±0.9)
**IOD**	2.7–4.2 (3.6±0.4)	1.7–2.8 (2.3±0.3)
**EW**	3.0–4.0 (3.5±0.3)	1.9–3.2 (2.6±0.3)
**IND**	1.8–3.7 (3.1±0.6)	1.1–2.9 (2.1±0.5)
**EN**	3.1–4.6 (4.0±0.5)	2.0–2.8 (2.4±0.2)
**TD**	1.5–3.0 (2.1±0.5)	1.0–1.7 (1.2±0.2)
**ED**	3.6–5.0 (4.4±0.5)	2.7–4.1 (3.4±0.4)

##### Coloration in life.

(based on digital photographs of the type specimens, Fig. 2).

Dorsum and flanks light brown (QCAZ 52473) to brown (QCAZ 52494, QCAZ 52512), with irregular dark brown marks bounded by dirty cream (QCAZ 52473), light brown (DHMECN 5236) or greenish cream (ZSFQ 1305) (Fig. 2); hidden surfaces of thighs brown splashed with dirty cream; groin with irregular yellowish marks; venter light gray (QCAZ 52474) or cream (QCAZ 52473) spotted to densely spotted (QCAZ 52474, DHMECN 5236, QCAZ 5212) with brown. Golden coppery iris with black reticulations and a reddish horizontal stripe (Fig. 2).

##### Distribution and natural history.

*Pristimantismallii* is only known from Fundación EcoMinga’s Río Zuñag Ecological Reserve, which is located in the southeastern buffer zone of the Llanganates National Park, in Baños, Río Negro, Tungurahua province, in the upper basin of the Pastaza River, on the east-central slope of the Andes in Ecuador. This locality comprises montane cloud forest (MAE 2012). The elevation range is 1300–2190 m above sea level.

All specimens were found on herbaceous and shrub vegetation inside mature forest, where they perched on herbs, shrubs, palms, ferns, bromeliads and Araceae between 100 and 450 cm above the ground. A couple in amplexus was found in January 2012, and the female deposited an egg clutch in a field bag, in the time passed between being collected in the field and reaching the base camp. Additionally, two couples in amplexus and an adult female were found in October 2017.

##### Etymology.

The new species is named in honor of the late Dr V. N. Mallikarjuna “Malli” Rao, of Wilmington, Delaware, USA. A winner of the Lavosier Medal at DuPont, he helped develop an environmentally safe alternative to the fluorocarbons that were depleting the ozone layer. His donation to EcoMinga in 2007 started the Río Zuñag Reserve, the type locality of *P.mallii*.

**Figure 7.**
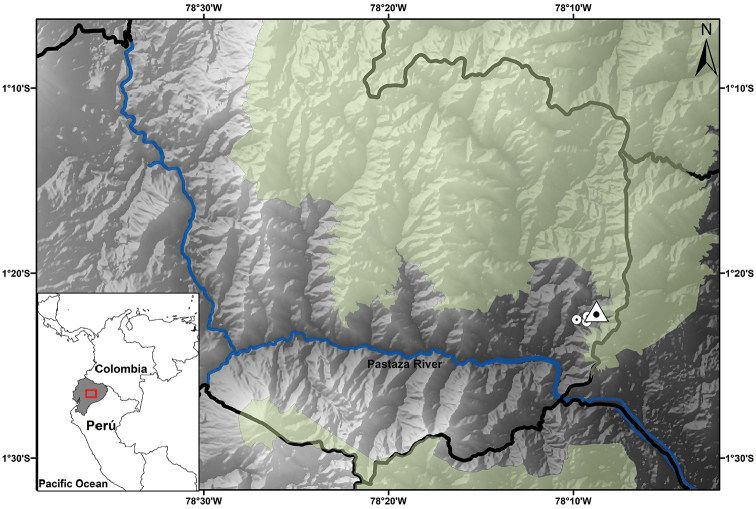
Map showing the four known localities for *Pristimantismallii* sp. n. Localities are based on type specimens deposited at the QCAZ, DHMECN and ZSFQ collections. Triangle represents the holotype locality; circles represents the paratypes localities.

## Discussion

*Pristimantismallii* is part of a clade of *Pristimantis* distributed in the Andes of central and southern Ecuador. The only non-Andean species of the group is *P.miktos*, which occurs in the Amazon basin below 300 m. We refrain from assigning the new species to a named species group. Most species groups in *Pristimantis* have been shown to be non-monophyletic, especially the large *P.unistrigatus* group (sensu Hedges et al. 2008). Of note, the clade presented in this paper is distributed in Andean forests, has a medium-sized SVL, lives in shrubby habits, and the majority of species have cryptic dorsal colorations and irregular diffuse flash marks on the hidden surfaces of the groin (Lynch 1979; Ortega-Andrade and Venegas 2014; Yánez-Muñoz et al. 2015; Ron et al. 2018).

The upper basin of the Pastaza River has proven to be a priority area for the conservation of *Pristimantis* due to its high alpha and beta diversity and high endemism (Reyes-Puig et al. 2013, 2014). In the last decade, nine species of *Pristimantis* have been described in this important region (Reyes-Puig et al. 2010, 2013, 2014, 2015; Yánez-Muñoz et al. 2010; Reyes-Puig and Yánez-Muñoz 2012). The discovery of *P.mallii*, represents the tenth species of *Pristimantis* discovered and described from the upper basin of the Pastaza River after one decade of herpetological research by the Instituto Nacional de Biodiversidad and the EcoMinga Foundation. These discoveries have helped biodiversity conservation outside government protected areas. The upper basin of the Pastaza River is a region with high diversity and endemism of several vertebrates (Reyes-Puig et al. 2013; Ríos-Alvear and Reyes-Puig 2015; Rodríguez-Galarza et al. 2017), but also of plants (e.g. Jost and Shepard 2017). The EcoMinga Foundation’s reserves function as a corridor between two large National Parks (i.e. Llanganates and Sangay) and contribute to the protection and connectivity of this important area. Coincidentally, this tenth new species of *Pristimantis* corresponds to ten years of herpetological research by EcoMinga which now manages ten ecological reserves. The total area protected within those reserves approximates 10,000 hectares.

## Supplementary Material

XML Treatment for Pristimantis mallii

## Appendix I

**Additional specimens examined**

***Pristimantiskichwarum*: Ecuador, Orellana**: QCAZ 22679, 22680, Parque Nacional Yasuní, 265 m; QCAZ 54894, Parque Nacional Yasuní, 290 m; QCAZ 20447, Parque Nacional Yasuní, vía Pompeya sur, 230 m; QCAZ 56572, Parque Nacional Ysauní, Tambococha, 203 m. ***Pristimantisluscombei*: Ecuador, Pastaza**: QCAZ 25456–25463, Kapawi Lodge, 239 m; QCAZ 54019, 54021– 54023, Curaray, 240 m; **Perú, Loreto**: QCAz 55640, 55649, 55650, Curaray Paiche, 200 m. ***Pristimantismarcoreyesi*: Ecuador, Tungurahua**: DHMECN 11343m Patate, Río Alisal, 3131 m; DHMECN 4816, 4819, 4822, Baños, Nahuazo Runtún, 2720 m. DHMECN 4825, 4830, San Antonio, Río Pucayacu, 2500 m; DHMECN 4818, 4823, 4824, 5084, Bosque Protector Cerro La Candelaria, 2700 m. ***Pristimantismiktos*: Ecuador, Morona Santiago**: QCAZ 53272, 53273, Tukupi, 211 m; Orellana: QCAZ 55445, Parque Nacional Yasuní, 175 m; QCAZ 49228– 49229, Parque Nacional Yasuní, 230 m; **Pastaza**: QCAZ 53581, 53582, Juyuintza, 200 m; QCAZ 54987, Lorocachi, 200 m; Perú, Loreto: QCAZ 55639, 55644, 55646, Curaray Paiche, 200 m. ***Pristimantisyanezi*: Ecuador, Napo**: QCAZ 46156, 46229, Tena, Carretera Salcedo Tena, 2253 m; QCAZ 46257–46259, Tena, Vía Salcedo Tena km 60, 2095 m; QCAZ 70089–70090, Tena Parque Nacional Llanganates, 2347 m; **Pastaza**: 66385, 66541, 66546, 66549, Mera, Reserva Comunitaria Ankaku, 2216 m.
